# *catena*-Poly[[bis­(dimethyl­ammonium) [cadmate(II)-bis­(μ-1,1′:4′,1′′-terphenyl-3,3′′-dicarboxyl­ato)]] dimethyl­formamide disolvate]

**DOI:** 10.1107/S1600536810051366

**Published:** 2010-12-11

**Authors:** Sang-Wook Park, Ja-Min Gu, Youngmee Kim, Seong Huh

**Affiliations:** a Department of Chemistry and Protein Research Center for Bio-Industry, Hankuk University of Foreign Studies, Yongin 449-791, Republic of Korea; b Department of Chemistry and Nano Science, Ewha Womans University, Seoul 120-750, Republic of Korea

## Abstract

In the title compound, {(C_2_H_8_N)_2_[Cd(C_20_H_12_O_4_)_2_]·2C_3_H_7_NO}_*n*_, the Cd^II^ ion lies on a twofold rotation axis and is in a distorted octa­hedral CdO_6_ environment, defined by four O atoms of two μ^2^-coordinated 1,1′:4′,1′′-terphenyl-3,3′′-dicarboxyl­ate (DCT) ligands and two O atoms of two μ^1^-coordinated DCT ligands. Both types of DCT ligands act as bridging, forming a one-dimensional polymeric structure propagating parallel to [10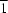].

## Related literature

For background information on metal-organic frameworks (MOFs), see: Li & Zhou (2009 ▶); Huh *et al.* (2009 ▶, 2010 ▶); Youm *et al.* (2004 ▶); Gu *et al.* (2010 ▶).
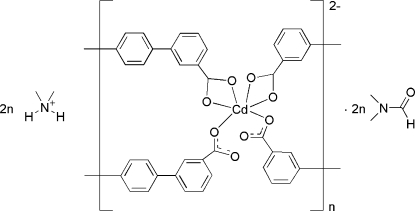


## Experimental

### 

#### Crystal data

(C_2_H_8_N)_2_[Cd(C_20_H_12_O_4_)_2_]·2C_3_H_7_NO
*M* *_r_* = 983.37
Monoclinic, 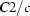

*a* = 28.525 (4) Å
*b* = 9.3267 (13) Å
*c* = 20.580 (3) Å
β = 114.752 (2)°
*V* = 4972.1 (11) Å^3^
*Z* = 4
Mo *K*α radiation
μ = 0.50 mm^−1^
*T* = 293 K
0.08 × 0.08 × 0.05 mm

#### Data collection

Bruker SMART CCD area-detector diffractometer
13515 measured reflections
4888 independent reflections
2861 reflections with *I* > 2σ(*I*)
*R*_int_ = 0.068

#### Refinement

*R*[*F* ^2^ > 2σ(*F* ^2^)] = 0.046
*wR*(*F* ^2^) = 0.116
*S* = 0.90
4888 reflections
298 parameters
H-atom parameters constrained
Δρ_max_ = 1.47 e Å^−3^
Δρ_min_ = −0.76 e Å^−3^

### 

Data collection: *SMART* (Bruker, 1997 ▶); cell refinement: *SAINT* (Bruker, 1997 ▶); data reduction: *SAINT*; program(s) used to solve structure: *SHELXS97* (Sheldrick, 2008 ▶); program(s) used to refine structure: *SHELXL97* (Sheldrick, 2008 ▶); molecular graphics: *SHELXTL* (Sheldrick, 2008 ▶); software used to prepare material for publication: *SHELXTL*.

## Supplementary Material

Crystal structure: contains datablocks I, global. DOI: 10.1107/S1600536810051366/lh5182sup1.cif

Structure factors: contains datablocks I. DOI: 10.1107/S1600536810051366/lh5182Isup2.hkl

Additional supplementary materials:  crystallographic information; 3D view; checkCIF report

## Figures and Tables

**Fig. 1.**
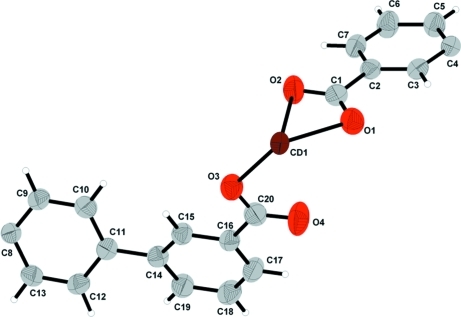
The asymmetric unit of the title compound's anion with labeling scheme. Displacement ellipsoids are shown at the 50% probability level.

**Fig. 2.**
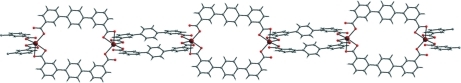
One-dimensional chain of the title compound. The dimethylammonium cations and DMF solvent molecules are omitted for clarity.

## supplementary crystallographic information

### Comment

The role of bridging ligands in the formation of structurally interesting metal-organic frameworks (MOFs) is of significant importance for the design of multi-functional MOFs and coordination polymers (Li & Zhou, 2009; Huh *et al.*, 2010; Huh *et al.*, 2009; Youm *et al.*, 2004). For instance, the reaction between a Zn(II) ion and a new *C*_2 h_-symmetric bridging ligand, 3,3"-dicarboxy-1,1':4',1"-terphenyl (DCT), in the presence of 1,4-diazacyclo[2,2,2]octane (DABCO) afforded a new nanoporous Zn-MOF containing DABCO ligands with an uncoordinated nitrogen atom towards one-dimensional channels. The resulting DABCO-functionalized Zn-MOF showed a better adsorption of CO_2_ over H_2_ and N_2_ with an exceptionally high CO_2_ adsorption enthalpy (Gu *et al.*, 2010).

To prepare new functional MOFs and coordination polymers, a reaction between Cd(NO_3_)_2_.H_2_O and the DCT ligand was investigated. A new one-dimensional coordination polymer, [H_2_N(CH_3_)_2_]_2_[Cd(DCT)_2_].2DMF (I), was obtained as colorless crystals and the crystal structure of (I) is reported herein. In the crystal structure of the title compound the Cd atom is coordinated by four oxygen atoms of two µ^2^-coordinated DCT ligands and two oxygen atoms of two µ^1^-coordinated DCT ligands (Fig. 1). Two DCT ligands bridging two Cd atoms and the title compound forms an extended one-dimensional coordination polymer (Fig. 2). The overall coordination environment of a Cd atom is a distorted octahedral geometry. The title compound possesses periodically arranged Cd^II^ ions with two negative charges per Cd center because of the charge mismatching between Cd^II^ ions and the DCT ligands. Therefore two dimethylammonium cations are required for charge balancing. In addtion there are two dimethylformamide solvent molecules in the formula unit.

### Experimental

The reaction mixture of Cd(NO_3_)_2_.H_2_O (30.8 mg, 0.1 mmol) and 3,3"-dicarboxy-1,1':4',1"-terphenyl (DCT, 32 mg, 0.1 mmol) in 10 ml of DMF was heated at 130 °C for 4 d. The resulting clear solution was storedat room temperature for few days gave colorless crystals.

### Refinement

H atoms were placed incalculated positions with C—H distances of 0.93 Å (phenyl) 0.96 Å (methyl) and N—H distances of 0.90 Å (ammonium). They were included in the refinement in riding-motion approximation with U_iso_(H) = 1.2U_eq_(C and N) and 1.5U_eq_(C).

### Crystal data

**Table:**

(C_2_H_8_N)_2_[Cd(C_20_H_12_O_4_)_2_]·2C_3_H_7_NO	*F*(000) = 2040
*M**_r_* = 983.37	*D*_x_ = 1.314 Mg m^−^^3^
Monoclinic, *C*2/*c*	Mo *K*α radiation, λ = 0.71073 Å
Hall symbol: -C 2yc	Cell parameters from 2049 reflections
*a* = 28.525 (4) Å	θ = 2.3–27.0°
*b* = 9.3267 (13) Å	µ = 0.50 mm^−^^1^
*c* = 20.580 (3) Å	*T* = 293 K
β = 114.752 (2)°	Block, colorless
*V* = 4972.1 (11) Å^3^	0.08 × 0.08 × 0.05 mm
*Z* = 4	

### Data collection

**Table:**

Bruker SMART CCD area-detector diffractometer	2861 reflections with *I* > 2σ(*I*)
Radiation source: fine-focus sealed tube	*R*_int_ = 0.068
graphite	θ_max_ = 26.0°, θ_min_ = 1.6°
φ and ω scans	*h* = −35→32
13515 measured reflections	*k* = −8→11
4888 independent reflections	*l* = −25→18

### Refinement

**Table:**

Refinement on *F*^2^	Primary atom site location: structure-invariant direct methods
Least-squares matrix: full	Secondary atom site location: difference Fourier map
*R*[*F*^2^ > 2σ(*F*^2^)] = 0.046	Hydrogen site location: inferred from neighbouring sites
*wR*(*F*^2^) = 0.116	H-atom parameters constrained
*S* = 0.90	*w* = 1/[σ^2^(*F*_o_^2^) + (0.0481*P*)^2^] where *P* = (*F*_o_^2^ + 2*F*_c_^2^)/3
4888 reflections	(Δ/σ)_max_ < 0.001
298 parameters	Δρ_max_ = 1.47 e Å^−^^3^
0 restraints	Δρ_min_ = −0.76 e Å^−^^3^

### Special details

**Table:**

Geometry. All e.s.d.'s (except the e.s.d. in the dihedral angle between two l.s. planes) are estimated using the full covariance matrix. The cell e.s.d.'s are taken into account individually in the estimation of e.s.d.'s in distances, angles and torsion angles; correlations between e.s.d.'s in cell parameters are only used when they are defined by crystal symmetry. An approximate (isotropic) treatment of cell e.s.d.'s is used for estimating e.s.d.'s involving l.s. planes.
Refinement. Refinement of *F*^2^ against ALL reflections. The weighted *R*-factor *wR* and goodness of fit *S* are based on *F*^2^, conventional *R*-factors *R* are based on *F*, with *F* set to zero for negative *F*^2^. The threshold expression of *F*^2^ > σ(*F*^2^) is used only for calculating *R*-factors(gt) *etc*. and is not relevant to the choice of reflections for refinement. *R*-factors based on *F*^2^ are statistically about twice as large as those based on *F*, and *R*- factors based on ALL data will be even larger.

### Fractional atomic coordinates and isotropic or equivalent isotropic displacement parameters (Å^2^)

**Table:**

	*x*	*y*	*z*	*U*_iso_*/*U*_eq_	
Cd1	1.0000	0.22586 (5)	0.7500	0.03992 (17)	
O1	1.08028 (11)	0.3209 (3)	0.77314 (16)	0.0572 (9)	
O2	1.01785 (11)	0.4040 (3)	0.67570 (16)	0.0621 (9)	
O3	0.95903 (10)	0.0827 (3)	0.65658 (14)	0.0505 (8)	
O4	1.03902 (12)	0.0114 (4)	0.68232 (16)	0.0719 (10)	
C1	1.06411 (17)	0.4007 (5)	0.7194 (2)	0.0449 (11)	
C2	1.10258 (15)	0.4955 (4)	0.7074 (2)	0.0389 (10)	
C3	1.15225 (14)	0.5095 (4)	0.7608 (2)	0.0390 (10)	
H3	1.1610	0.4619	0.8040	0.047*	
C4	1.18932 (14)	0.5936 (4)	0.7510 (2)	0.0382 (10)	
C5	1.17464 (16)	0.6611 (5)	0.6854 (2)	0.0482 (12)	
H5	1.1987	0.7164	0.6770	0.058*	
C6	1.12548 (16)	0.6485 (5)	0.6323 (2)	0.0534 (12)	
H6	1.1167	0.6951	0.5888	0.064*	
C7	1.08924 (16)	0.5672 (5)	0.6433 (2)	0.0471 (11)	
H7	1.0558	0.5605	0.6077	0.056*	
C8	0.74239 (14)	−0.1084 (4)	0.3083 (2)	0.0377 (10)	
C9	0.76565 (15)	0.0007 (5)	0.3571 (2)	0.0452 (11)	
H9	0.7467	0.0827	0.3560	0.054*	
C10	0.81649 (16)	−0.0094 (5)	0.4076 (2)	0.0465 (11)	
H10	0.8308	0.0657	0.4395	0.056*	
C11	0.84628 (15)	−0.1288 (5)	0.4114 (2)	0.0379 (10)	
C12	0.82222 (15)	−0.2418 (5)	0.3648 (2)	0.0443 (11)	
H12	0.8404	−0.3260	0.3677	0.053*	
C13	0.77171 (15)	−0.2300 (5)	0.3145 (2)	0.0463 (11)	
H13	0.7569	−0.3066	0.2837	0.056*	
C14	0.90158 (15)	−0.1368 (4)	0.4619 (2)	0.0385 (10)	
C15	0.92021 (15)	−0.0652 (4)	0.5273 (2)	0.0408 (10)	
H15	0.8975	−0.0131	0.5400	0.049*	
C16	0.97212 (15)	−0.0706 (4)	0.5738 (2)	0.0396 (10)	
C17	1.00572 (16)	−0.1481 (5)	0.5552 (2)	0.0506 (12)	
H17	1.0406	−0.1523	0.5861	0.061*	
C18	0.98748 (16)	−0.2195 (5)	0.4906 (2)	0.0541 (12)	
H18	1.0102	−0.2724	0.4782	0.065*	
C19	0.93633 (16)	−0.2132 (5)	0.4445 (2)	0.0480 (11)	
H19	0.9248	−0.2609	0.4009	0.058*	
C20	0.99169 (17)	0.0116 (5)	0.6434 (2)	0.0473 (11)	
N21	0.1478 (2)	0.4189 (6)	0.9596 (2)	0.0814 (14)	
O21	0.16600 (16)	0.1981 (4)	0.9324 (2)	0.0925 (13)	
C21	0.1350 (2)	0.2929 (7)	0.9302 (3)	0.0737 (16)	
H21	0.1000	0.2727	0.9057	0.088*	
C22	0.2015 (3)	0.4610 (9)	0.9988 (4)	0.146 (3)	
H22A	0.2235	0.3826	0.9996	0.220*	
H22B	0.2077	0.4863	1.0469	0.220*	
H22C	0.2088	0.5420	0.9757	0.220*	
C23	0.1082 (3)	0.5226 (8)	0.9496 (3)	0.126 (3)	
H23A	0.1143	0.6068	0.9275	0.189*	
H23B	0.1088	0.5475	0.9952	0.189*	
H23C	0.0752	0.4828	0.9196	0.189*	
N31	0.12647 (13)	1.0202 (4)	0.80646 (18)	0.0537 (10)	
H31A	0.1302	1.0770	0.8436	0.064*	
H31B	0.0943	1.0331	0.7724	0.064*	
C31	0.16355 (19)	1.0657 (7)	0.7781 (3)	0.0912 (19)	
H31C	0.1980	1.0579	0.8149	0.137*	
H31D	0.1568	1.1634	0.7623	0.137*	
H31E	0.1601	1.0055	0.7385	0.137*	
C32	0.1320 (2)	0.8719 (6)	0.8299 (3)	0.101 (2)	
H32A	0.1310	0.8107	0.7919	0.152*	
H32B	0.1042	0.8471	0.8426	0.152*	
H32C	0.1643	0.8599	0.8708	0.152*	

### Atomic displacement parameters (Å^2^)

**Table:**

	*U*^11^	*U*^22^	*U*^33^	*U*^12^	*U*^13^	*U*^23^
Cd1	0.0305 (2)	0.0450 (3)	0.0417 (3)	0.000	0.01258 (19)	0.000
O1	0.0486 (19)	0.058 (2)	0.060 (2)	−0.0061 (16)	0.0182 (16)	0.0137 (17)
O2	0.0367 (18)	0.067 (2)	0.072 (2)	−0.0066 (16)	0.0126 (17)	0.0110 (17)
O3	0.0426 (18)	0.061 (2)	0.0476 (18)	0.0010 (15)	0.0182 (15)	−0.0085 (15)
O4	0.0410 (19)	0.101 (3)	0.055 (2)	0.0050 (19)	0.0016 (16)	−0.0156 (19)
C1	0.042 (3)	0.041 (3)	0.051 (3)	0.002 (2)	0.019 (2)	−0.003 (2)
C2	0.037 (2)	0.035 (3)	0.047 (3)	0.0023 (19)	0.020 (2)	0.002 (2)
C3	0.038 (2)	0.038 (3)	0.040 (2)	0.005 (2)	0.015 (2)	0.0036 (19)
C4	0.033 (2)	0.037 (3)	0.044 (3)	0.0013 (19)	0.015 (2)	0.000 (2)
C5	0.040 (3)	0.053 (3)	0.053 (3)	−0.004 (2)	0.020 (2)	0.014 (2)
C6	0.043 (3)	0.064 (3)	0.048 (3)	0.002 (2)	0.014 (2)	0.016 (2)
C7	0.035 (2)	0.048 (3)	0.051 (3)	0.004 (2)	0.011 (2)	0.007 (2)
C8	0.035 (2)	0.036 (3)	0.042 (2)	0.000 (2)	0.016 (2)	0.001 (2)
C9	0.041 (3)	0.041 (3)	0.052 (3)	0.006 (2)	0.017 (2)	−0.006 (2)
C10	0.046 (3)	0.042 (3)	0.045 (3)	−0.002 (2)	0.013 (2)	−0.006 (2)
C11	0.039 (2)	0.042 (3)	0.034 (2)	0.002 (2)	0.0158 (19)	0.002 (2)
C12	0.044 (2)	0.040 (3)	0.048 (3)	0.006 (2)	0.018 (2)	−0.002 (2)
C13	0.045 (2)	0.040 (3)	0.052 (3)	−0.003 (2)	0.018 (2)	−0.011 (2)
C14	0.040 (2)	0.040 (3)	0.034 (2)	0.001 (2)	0.014 (2)	0.001 (2)
C15	0.037 (2)	0.042 (3)	0.044 (3)	0.001 (2)	0.018 (2)	0.002 (2)
C16	0.038 (2)	0.040 (3)	0.038 (2)	0.002 (2)	0.012 (2)	−0.001 (2)
C17	0.038 (3)	0.057 (3)	0.051 (3)	0.009 (2)	0.012 (2)	−0.001 (2)
C18	0.048 (3)	0.056 (3)	0.060 (3)	0.010 (2)	0.024 (2)	−0.008 (3)
C19	0.045 (3)	0.051 (3)	0.045 (3)	0.002 (2)	0.015 (2)	−0.010 (2)
C20	0.047 (3)	0.046 (3)	0.045 (3)	−0.002 (2)	0.015 (2)	0.003 (2)
N21	0.092 (4)	0.076 (4)	0.074 (3)	−0.010 (3)	0.034 (3)	−0.021 (3)
O21	0.095 (3)	0.086 (3)	0.078 (3)	0.011 (2)	0.018 (2)	−0.011 (2)
C21	0.090 (4)	0.067 (4)	0.054 (3)	−0.016 (4)	0.020 (3)	−0.010 (3)
C22	0.108 (6)	0.159 (8)	0.171 (7)	−0.055 (5)	0.057 (5)	−0.080 (6)
C23	0.151 (7)	0.092 (6)	0.130 (6)	0.007 (5)	0.054 (5)	−0.012 (4)
N31	0.046 (2)	0.060 (3)	0.049 (2)	0.010 (2)	0.0142 (19)	−0.0055 (19)
C31	0.069 (4)	0.108 (5)	0.113 (5)	0.000 (3)	0.055 (4)	−0.002 (4)
C32	0.134 (6)	0.064 (4)	0.127 (5)	0.021 (4)	0.075 (5)	0.014 (4)

### Geometric parameters (Å, °)

**Table:**

Cd1—O3	2.228 (3)	C12—H12	0.9300
Cd1—O3^i^	2.228 (3)	C13—H13	0.9300
Cd1—O1	2.312 (3)	C14—C19	1.383 (5)
Cd1—O1^i^	2.312 (3)	C14—C15	1.394 (5)
Cd1—O2	2.451 (3)	C15—C16	1.387 (5)
Cd1—O2^i^	2.451 (3)	C15—H15	0.9300
Cd1—C1^i^	2.714 (4)	C16—C17	1.376 (5)
Cd1—C1	2.714 (4)	C16—C20	1.510 (6)
O1—C1	1.250 (5)	C17—C18	1.380 (6)
O2—C1	1.248 (4)	C17—H17	0.9300
O3—C20	1.262 (5)	C18—C19	1.369 (5)
O4—C20	1.249 (5)	C18—H18	0.9300
C1—C2	1.508 (6)	C19—H19	0.9300
C2—C7	1.383 (5)	N21—C21	1.302 (6)
C2—C3	1.389 (5)	N21—C23	1.434 (7)
C3—C4	1.397 (5)	N21—C22	1.455 (7)
C3—H3	0.9300	O21—C21	1.238 (6)
C4—C5	1.386 (5)	C21—H21	0.9300
C4—C8^ii^	1.485 (5)	C22—H22A	0.9600
C5—C6	1.375 (5)	C22—H22B	0.9600
C5—H5	0.9300	C22—H22C	0.9600
C6—C7	1.375 (5)	C23—H23A	0.9600
C6—H6	0.9300	C23—H23B	0.9600
C7—H7	0.9300	C23—H23C	0.9600
C8—C13	1.383 (5)	N31—C32	1.452 (6)
C8—C9	1.388 (5)	N31—C31	1.469 (6)
C8—C4^iii^	1.485 (5)	N31—H31A	0.9000
C9—C10	1.389 (5)	N31—H31B	0.9000
C9—H9	0.9300	C31—H31C	0.9600
C10—C11	1.383 (5)	C31—H31D	0.9600
C10—H10	0.9300	C31—H31E	0.9600
C11—C12	1.396 (5)	C32—H32A	0.9600
C11—C14	1.484 (5)	C32—H32B	0.9600
C12—C13	1.383 (5)	C32—H32C	0.9600
			
O3—Cd1—O3^i^	106.36 (15)	C10—C11—C14	121.9 (4)
O3—Cd1—O1	121.60 (10)	C12—C11—C14	121.0 (4)
O3^i^—Cd1—O1	86.31 (11)	C13—C12—C11	120.9 (4)
O3—Cd1—O1^i^	86.31 (11)	C13—C12—H12	119.6
O3^i^—Cd1—O1^i^	121.60 (10)	C11—C12—H12	119.6
O1—Cd1—O1^i^	134.91 (16)	C12—C13—C8	122.3 (4)
O3—Cd1—O2	92.22 (10)	C12—C13—H13	118.9
O3^i^—Cd1—O2	140.68 (10)	C8—C13—H13	118.9
O1—Cd1—O2	54.70 (10)	C19—C14—C15	118.1 (4)
O1^i^—Cd1—O2	93.34 (10)	C19—C14—C11	120.8 (4)
O3—Cd1—O2^i^	140.68 (10)	C15—C14—C11	121.0 (4)
O3^i^—Cd1—O2^i^	92.22 (10)	C16—C15—C14	121.0 (4)
O1—Cd1—O2^i^	93.34 (11)	C16—C15—H15	119.5
O1^i^—Cd1—O2^i^	54.69 (10)	C14—C15—H15	119.5
O2—Cd1—O2^i^	94.67 (15)	C17—C16—C15	119.6 (4)
O3—Cd1—C1^i^	113.54 (12)	C17—C16—C20	120.4 (4)
O3^i^—Cd1—C1^i^	108.72 (12)	C15—C16—C20	120.0 (4)
O1—Cd1—C1^i^	115.31 (12)	C16—C17—C18	119.7 (4)
O1^i^—Cd1—C1^i^	27.33 (10)	C16—C17—H17	120.1
O2—Cd1—C1^i^	94.22 (11)	C18—C17—H17	120.1
O2^i^—Cd1—C1^i^	27.36 (10)	C19—C18—C17	120.6 (4)
O3—Cd1—C1	108.72 (12)	C19—C18—H18	119.7
O3^i^—Cd1—C1	113.54 (12)	C17—C18—H18	119.7
O1—Cd1—C1	27.34 (10)	C18—C19—C14	121.0 (4)
O1^i^—Cd1—C1	115.31 (12)	C18—C19—H19	119.5
O2—Cd1—C1	27.36 (10)	C14—C19—H19	119.5
O2^i^—Cd1—C1	94.22 (11)	O4—C20—O3	124.1 (4)
C1^i^—Cd1—C1	106.12 (18)	O4—C20—C16	118.6 (4)
C1—O1—Cd1	94.5 (3)	O3—C20—C16	117.2 (4)
C1—O2—Cd1	88.1 (3)	C21—N21—C23	119.5 (6)
C20—O3—Cd1	109.4 (3)	C21—N21—C22	121.7 (6)
O2—C1—O1	122.7 (4)	C23—N21—C22	118.7 (6)
O2—C1—C2	119.3 (4)	O21—C21—N21	124.9 (6)
O1—C1—C2	118.0 (4)	O21—C21—H21	117.5
O2—C1—Cd1	64.5 (2)	N21—C21—H21	117.5
O1—C1—Cd1	58.1 (2)	N21—C22—H22A	109.5
C2—C1—Cd1	176.1 (3)	N21—C22—H22B	109.5
C7—C2—C3	119.4 (4)	H22A—C22—H22B	109.5
C7—C2—C1	120.8 (4)	N21—C22—H22C	109.5
C3—C2—C1	119.8 (4)	H22A—C22—H22C	109.5
C2—C3—C4	121.4 (4)	H22B—C22—H22C	109.5
C2—C3—H3	119.3	N21—C23—H23A	109.5
C4—C3—H3	119.3	N21—C23—H23B	109.5
C5—C4—C3	117.2 (4)	H23A—C23—H23B	109.5
C5—C4—C8^ii^	121.5 (4)	N21—C23—H23C	109.5
C3—C4—C8^ii^	121.3 (4)	H23A—C23—H23C	109.5
C6—C5—C4	121.8 (4)	H23B—C23—H23C	109.5
C6—C5—H5	119.1	C32—N31—C31	114.3 (4)
C4—C5—H5	119.1	C32—N31—H31A	108.7
C7—C6—C5	120.2 (4)	C31—N31—H31A	108.7
C7—C6—H6	119.9	C32—N31—H31B	108.7
C5—C6—H6	119.9	C31—N31—H31B	108.7
C6—C7—C2	119.9 (4)	H31A—N31—H31B	107.6
C6—C7—H7	120.1	N31—C31—H31C	109.5
C2—C7—H7	120.1	N31—C31—H31D	109.5
C13—C8—C9	116.6 (4)	H31C—C31—H31D	109.5
C13—C8—C4^iii^	121.7 (4)	N31—C31—H31E	109.5
C9—C8—C4^iii^	121.7 (4)	H31C—C31—H31E	109.5
C8—C9—C10	121.6 (4)	H31D—C31—H31E	109.5
C8—C9—H9	119.2	N31—C32—H32A	109.5
C10—C9—H9	119.2	N31—C32—H32B	109.5
C11—C10—C9	121.5 (4)	H32A—C32—H32B	109.5
C11—C10—H10	119.3	N31—C32—H32C	109.5
C9—C10—H10	119.3	H32A—C32—H32C	109.5
C10—C11—C12	117.0 (4)	H32B—C32—H32C	109.5
			
O3—Cd1—O1—C1	68.3 (3)	C2—C3—C4—C5	−0.8 (6)
O3^i^—Cd1—O1—C1	175.3 (3)	C2—C3—C4—C8^ii^	180.0 (4)
O1^i^—Cd1—O1—C1	−52.1 (2)	C3—C4—C5—C6	1.1 (6)
O2—Cd1—O1—C1	0.6 (2)	C8^ii^—C4—C5—C6	−179.6 (4)
O2^i^—Cd1—O1—C1	−92.7 (3)	C4—C5—C6—C7	0.0 (7)
C1^i^—Cd1—O1—C1	−75.8 (3)	C5—C6—C7—C2	−1.4 (7)
O3—Cd1—O2—C1	−128.6 (3)	C3—C2—C7—C6	1.7 (6)
O3^i^—Cd1—O2—C1	−9.1 (3)	C1—C2—C7—C6	−177.4 (4)
O1—Cd1—O2—C1	−0.6 (2)	C13—C8—C9—C10	2.6 (6)
O1^i^—Cd1—O2—C1	145.0 (3)	C4^iii^—C8—C9—C10	−175.6 (4)
O2^i^—Cd1—O2—C1	90.1 (3)	C8—C9—C10—C11	0.1 (6)
C1^i^—Cd1—O2—C1	117.6 (3)	C9—C10—C11—C12	−3.3 (6)
O3^i^—Cd1—O3—C20	−64.4 (3)	C9—C10—C11—C14	175.9 (4)
O1—Cd1—O3—C20	31.4 (3)	C10—C11—C12—C13	3.7 (6)
O1^i^—Cd1—O3—C20	173.7 (3)	C14—C11—C12—C13	−175.5 (4)
O2—Cd1—O3—C20	80.5 (3)	C11—C12—C13—C8	−1.0 (6)
O2^i^—Cd1—O3—C20	−179.4 (2)	C9—C8—C13—C12	−2.2 (6)
C1^i^—Cd1—O3—C20	176.1 (3)	C4^iii^—C8—C13—C12	176.1 (4)
C1—Cd1—O3—C20	58.2 (3)	C10—C11—C14—C19	−148.1 (4)
Cd1—O2—C1—O1	1.1 (4)	C12—C11—C14—C19	31.1 (6)
Cd1—O2—C1—C2	−179.3 (3)	C10—C11—C14—C15	30.5 (6)
Cd1—O1—C1—O2	−1.2 (5)	C12—C11—C14—C15	−150.4 (4)
Cd1—O1—C1—C2	179.2 (3)	C19—C14—C15—C16	−0.1 (6)
O3—Cd1—C1—O2	55.5 (3)	C11—C14—C15—C16	−178.7 (4)
O3^i^—Cd1—C1—O2	173.7 (2)	C14—C15—C16—C17	−0.2 (6)
O1—Cd1—C1—O2	178.9 (4)	C14—C15—C16—C20	178.1 (4)
O1^i^—Cd1—C1—O2	−39.3 (3)	C15—C16—C17—C18	0.0 (7)
O2^i^—Cd1—C1—O2	−92.0 (3)	C20—C16—C17—C18	−178.3 (4)
C1^i^—Cd1—C1—O2	−66.9 (2)	C16—C17—C18—C19	0.5 (7)
O3—Cd1—C1—O1	−123.3 (3)	C17—C18—C19—C14	−0.8 (7)
O3^i^—Cd1—C1—O1	−5.2 (3)	C15—C14—C19—C18	0.6 (6)
O1^i^—Cd1—C1—O1	141.8 (2)	C11—C14—C19—C18	179.2 (4)
O2—Cd1—C1—O1	−178.9 (4)	Cd1—O3—C20—O4	7.4 (5)
O2^i^—Cd1—C1—O1	89.1 (3)	Cd1—O3—C20—C16	−168.7 (3)
C1^i^—Cd1—C1—O1	114.2 (3)	C17—C16—C20—O4	2.3 (6)
O2—C1—C2—C7	−10.8 (6)	C15—C16—C20—O4	−176.0 (4)
O1—C1—C2—C7	168.7 (4)	C17—C16—C20—O3	178.6 (4)
O2—C1—C2—C3	170.1 (4)	C15—C16—C20—O3	0.4 (6)
O1—C1—C2—C3	−10.3 (6)	C23—N21—C21—O21	176.3 (6)
C7—C2—C3—C4	−0.6 (6)	C22—N21—C21—O21	0.3 (9)
C1—C2—C3—C4	178.5 (4)		

Symmetry codes: (i) −*x*+2, *y*, −*z*+3/2; (ii) *x*+1/2, −*y*+1/2, *z*+1/2; (iii) *x*−1/2, −*y*+1/2, *z*−1/2.
